# Genome-Wide Association Study and Candidate Gene Identification for Girth Traits in Rubber Tree

**DOI:** 10.3390/plants14162460

**Published:** 2025-08-08

**Authors:** Wenxiu Li, Zishan Zhang, Huan Ouyang, Hualin Zhang, Han Cheng, Xiaofei Zhang, Xinsheng Gao, Junjun He, Qing Yan, Yana Ye, Yingtao Yi, Pingsheng Li, Ping Luo, Ruihong Xie

**Affiliations:** 1 Zhanjiang Experimental Station, Chinese Academy of Tropical Agricultural Sciences, Zhanjiang 524013, China; liwenxiudeyouxiang@126.com (W.L.); 62551912@163.com (H.O.); happy_zhl1985@163.com (H.Z.); zjzyq@catas.cn (Q.Y.); yyn_ana@163.com (Y.Y.); 2 School of Life Science and Technology, Lingnan Normal University, Zhanjiang 524048, China; 17827786883@163.com; 3 Rubber Research Institute, Chinese Academy of Tropical Agricultural Science, Haikou 571101, China; forcheng@gmail.com (H.C.); xjszhxf@163.com (X.Z.); hagaoxs@163.com (X.G.); 4 South Subtropical Crop Research Institute, Chinese Academy of Tropical Agricultural Sciences, Zhanjiang 524013, China; hbj46@163.com; 5 College of Tropical Crops, Yunnan Agricultural University, Pu’er 665099, China; 13618795719@163.com (Y.Y.); 18314051395@163.com (P.L.)

**Keywords:** rubber tree, growth traits, genome-wide association analysis, candidate genes

## Abstract

As a key tropical economic tree species, the girth of the rubber tree (*Hevea brasiliensis*) not only reflects its growth rate and timber yield but also determines tapping schedules and non-productive periods. This trait critically influences both the species’ economic value and latex production potential. Despite recent advances in genetic analyses of girth driven by genomic technologies, the number of identified key genes remains insufficient to support molecular breeding programs. This study focuses on 138 samples of rubber tree natural accessions, integrating phenotypic data analysis, population genetic structure analysis, and genome-wide association analysis (GWAS) to identify genetic loci and candidate genes associated with girth. Population stratification divides the tested accessions into four genetic groups: Groups Ⅰ and Ⅳ exhibit high genetic purity, while Groups Ⅱ and Ⅲ display hybrid characteristics. GWAS based on a mixed linear model detects 7 and 23 SNPs significantly associated with girth at *p* = 4.4 × 10^−8^ and *p* = 2.22 × 10^−7^, respectively. The most significant SNP is located at position 44994744 on chromosome CM021229.1. Under the highly significant association threshold, 27 candidate genes were identified, 4 of which are directly related to girth. Gene Ontology (GO) annotation of these 27 candidate genes reveals their primary involvement in metabolic regulation, signal transduction, and cell component construction. Kyoto Encyclopedia of Genes and Genomes (KEGG) analysis shows they are primarily enriched in the “aminoacyl-tRNA biosynthesis” and “glycolysis/gluconeogenesis” pathways. These findings provide significant theoretical support for genetic enhancement and mechanistic analysis of rubber tree growth traits. They reveal novel SNP markers and genes that complement existing genetic resources, refining breeding strategies for elite genotype selection and ultimately contributing to enhanced rubber production.

## 1. Introduction

Rubber tree (*Hevea brasiliensis*), a perennial tropical rainforest species belonging to the genus *Hevea* in the family Euphorbiaceae, is native to the Amazon Basin in Brazil. As the primary global source of natural rubber, it possesses biologically irreplaceable properties and holds significant economic and strategic value. The natural rubber it produces serves as a core industrial material and strategic resource indispensable for economic development [1]. Stem girth is a key indicator of growth performance and timber yield in rubber trees: fast-growing individuals accumulate more xylem tissue, resulting in higher timber output over the same period. Despite a lifespan of 30–40 years, rubber trees only begin to be tapped after 7–8 years, leading to a prolonged non-productive phase and high initial planting costs. Girth directly determines a tree’s readiness for tapping, as sufficient stem thickness and well-developed internal latex vessel systems are essential for stable latex production [2]. Thus, identifying girth-related genes and developing fast-growing varieties through molecular breeding is crucial. This approach can shorten the non-productive period, accelerate the onset of tapping, and enhance the economic benefits for rubber farmers.

In recent years, advancements in genomic technologies have enabled researchers to achieve significant progress in the genetic analysis of rubber tree girth through multi-omics integrative analyses. Chanroj et al. [3] were the first to construct a genome-wide association map of rubber tree latex yield and girth traits under suboptimal habitats, revealing the linkage disequilibrium (LD) decay characteristics similar to those of cross-pollinated species. Zhang et al. [4] identified eight girth-related quantitative trait loci (QTLs), among which *HbGID1* was recognized as a promising candidate gene. Bhusudsawang et al. [5] used an intron length polymorphism (ILP) marker-based approach to identify COBL4 as a candidate gene associated with rubber tree girth growth. Francisco et al. [6] integrated GWAS and RNA-Seq technologies to explore core genes related to rubber tree growth, uncovering several plant growth-associated genes (e.g., SBT4.6, GK1). Souza et al. [7] detected seven QTLs linked to rubber tree girth growth under suboptimal climatic conditions in Brazil. Conson et al. [8] identified 53 QTLs significantly associated with girth under suboptimal temperature and humidity conditions. Liang et al. [9] identified 31 candidate genes significantly associated with girth via GWAS, providing important insights for genetic analysis and breeding of related traits in Para rubber trees. However, rubber tree growth traits are controlled by multiple genes, and the contribution rate of a single gene or individual marker is typically limited. Furthermore, the development of practical molecular markers generally requires validation using large-scale datasets. The key genes identified in existing studies are still insufficient, with only a few genes (such as HbGID1, COBL4, and GK1) having been verified, making it challenging to meet the demands of molecular breeding.

GWAS, a core technology for plant genetic analysis, has demonstrated unique advantages in identifying genes associated with important traits. Compared to conventional quantitative trait locus (QTL) linkage analysis, GWAS has several key benefits [10]: it enables precise trait localization using high-density genome-wide molecular markers, with significantly improved resolution [11]. It overcomes the limitation of multi-allele detection and allows parallel analysis of multiple allelic sites [12]. GWAS can simultaneously and extensively mine alleles significantly associated with multiple trait variations across the genome [13].

Currently, GWAS has been widely used to identify potential agronomic trait-related genes in many species, such as sesame [14], cucumber [15], wheat [16], rice [17], and rubber tree [18,19]. In this study, we used 138 natural accessions (comprising 55 collected accessions, 76 hybrid-derived accessions, and 7 internationally introduced accessions) of rubber trees as materials and employed high-throughput sequencing technology to obtain whole-genome sequences. We then performed population structure analysis and GWAS, leveraged the LD relationship between genetic variations and functional genes to identify candidate genes significantly associated with girth, and annotated the candidate genes based on the GO and KEGG databases. The research results will provide a reference for subsequent exploration of rubber tree growth trait improvement and selection of superior genotypes.

## 2. Results

### 2.1. Phenotypic Data Analysis

The girth of 138 rubber tree accessions was measured and analyzed. As shown in Table 1, significant phenotypic variation in girth was observed within the population, with statistical parameters including a mean of 76.13 cm, standard deviation of 15.57, standard error of 1.33, minimum of 48.5 cm, maximum of 142 cm, range of 93.5 cm, skewness of 1.05, and kurtosis of 1.62. This phenotypic variation provides a solid foundation for subsequent GWAS.

**Figure 1:**
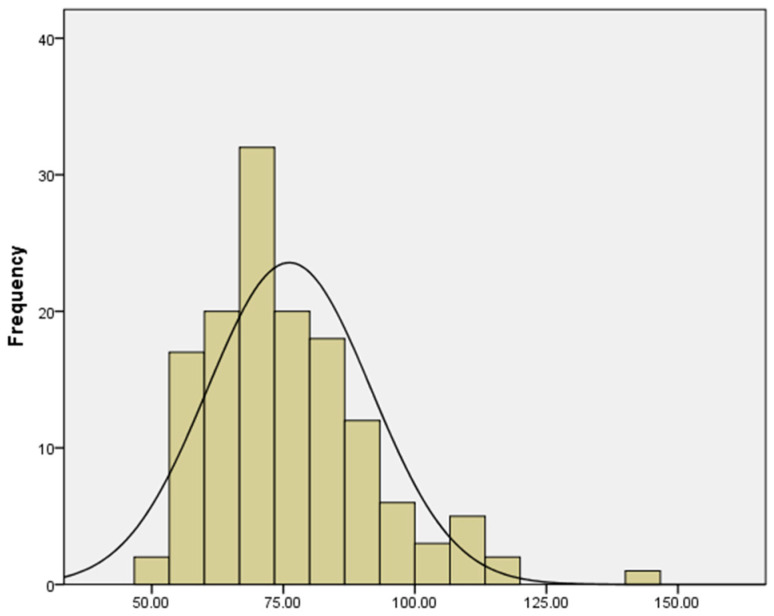
Trait frequency histogram. KS test: *p* = 0.143 > 0.05.

The normality test results (*p* = 0.143 > 0.05) indicated that girth was normally distributed. The distribution pattern of each phenotypic value is shown in the following figure (Figure 1).

### 2.2. Population Stratification Analysis

**Figure 2:**
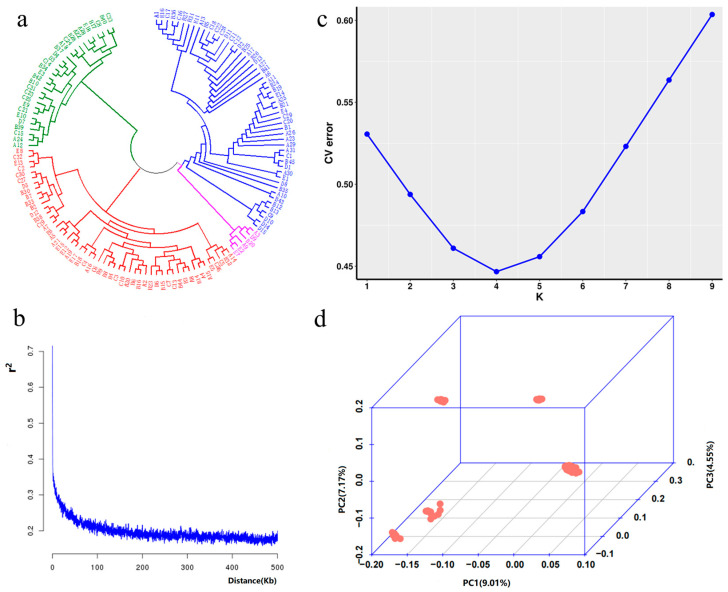
Cluster analysis chart: (**a**) NJ tree of 138 rubber tree accessions; Green represents Group Ⅰ, orange represents Group ⅠⅠ, blue represents Group ⅠⅠⅠ, and pink represents Group Ⅳ. (**b**) LD decay chart; (**c**) cross-validation error rate line chart; (**d**) 3D PCA clustering chart.

A multidimensional population genetic analysis of the whole-genome SNP markers of 138 rubber tree accessions revealed significant genetic diversity. The neighbor-joining (NJ) tree based on the p-distance model divided the accessions into four main groups (Figure 2a): Group Ⅰ (28 accessions), Group Ⅱ (51 accessions), Group Ⅲ (53 accessions), and Group Ⅳ (6 accessions).

Linkage disequilibrium (LD) analysis showed that genome-wide LD values (r^2^) decreased exponentially with physical distance (Figure 2b). At a physical distance of 50 kb, the average r^2^ dropped below 0.2, aligning with the low LD levels typical of the outcrossing rubber tree. Admixture analysis found the cross-validation error minimized at K = 4 (0.44), indicating four major genetic components (Figure 2c).

**Figure 3:**
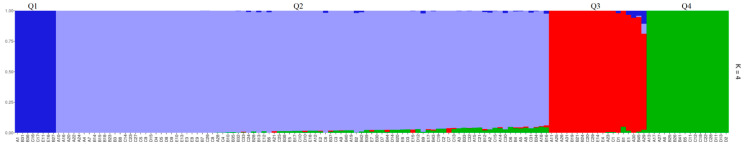
Bar chart of genetic composition of samples.

Genetic component distribution revealed high genetic purity in Groups Ⅰ and Ⅳ, while Groups Ⅱ and Ⅲ showed mixed genetic features (Figure 3). The PCA results were consistent with this, with the first three principal components (PC1 = 9.01%, PC2 = 7.17%, PC3 = 4.95%) explaining 21.13% of genetic variation (Figure 2d). PC1 clearly separated Groups Ⅰ and Ⅳ, while PC2 reflected the differentiation between Groups Ⅱ and Ⅲ.

### 2.3. Genome-Wide Association Analysis

**Figure 4:**
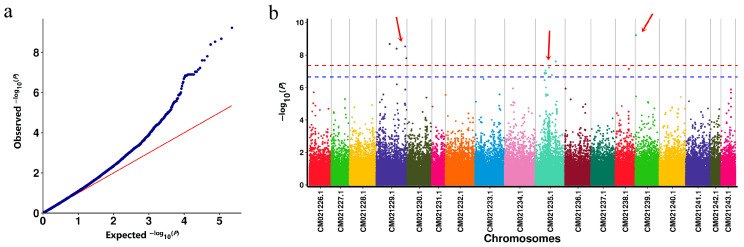
Linkage disequilibrium analysis: (**a**) MLM (K), Q-Q plot; (**b**) MLM (K), Manhattan plot. The blue horizontal dashed line represents the position of *p* = 2.22 × 10^−7^ after taking −log10; the red horizontal dashed line represents the position of *p* = 4.4 × 10^−8^ after taking −log10. The red arrow represents the significance marker.

Using girth phenotype data and filtered SNP markers, a GWAS was performed via a mixed linear model (MLM, incorporating kinship matrix K). The Manhattan plot and Q-Q plot (Figure 4a) revealed 7 SNPs reaching genome-wide significance at the *p* = 4.4 × 10^−8^ threshold and 23 SNPs at the *p* = 2.22 × 10^−7^ level (see Supplementary Table S1), both associated with stem girth. These SNPs were mapped to chromosomes CM021229.1, CM021235.1, and CM021239.1 (Table 2). The most strongly associated SNP was at position 44994744 on CM021229.1 (*p* = 2.08 × 10^−9^, R^2^ = 0.254). Nearby candidate genes include GH714_028663 (encoding SGS3) and GH714_028652 (encoding UGT83A1).

### 2.4. Candidate Gene Identification and Enrichment Analysis

**Figure 5:**
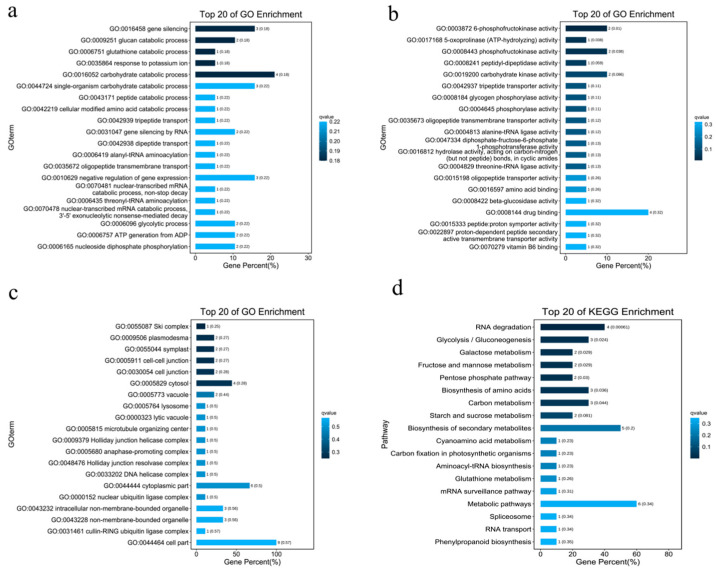
Enrichment bar charts: (**a**) bar chart of biological process GO enrichment; (**b**) bar chart of molecular function GO enrichment; (**c**) bar chart of cellular component GO enrichment; (**d**) bar chart of KO enrichment.

This study identified 27 candidate genes based on GWAS significant loci, four of which (GH714_035173/PHS2, GH714_020134/PFK3, GH714_020079/PFK4, GH714_035167/BACOVA_02659) are linked to girth. Enrichment analysis revealed their roles: PFK3 and PFK4 regulate sugar metabolism for energy and monosaccharide supply for cell wall synthesis; POPTRDRAFT_821063 ensures protein production for cell division; BACOVA_02659 and UGT83A1 influence cell wall by regulating lignin and cellulose metabolism; NPF8.1 and RGA3 control cell elongation and division through hormonal signaling pathways [20]; OXP1 maintains cellular redox balance, facilitating continuous cell division [21]; SGS3 affects cell elongation by regulating RNA stability; UGT83A1 may participate in secondary metabolite glycosylation related to lignin synthesis. Among these, PFK3/PFK4 [22], PHS2 [23], and BACOVA_02659 [24] have been studied in plants like *Arabidopsis* and maize for their roles in energy metabolism, cell wall synthesis, hormone transport, and oxidative protection, supporting girth growth. GO annotations indicate that candidate genes mainly participate in metabolic regulation, signal transduction, and cell component construction. GO enrichment analysis shows significant enrichment in biological processes like “metabolic processes” (GO:0008152, 18 genes), “protein phosphorylation” (GO:0006468, 6 genes), and “RNA processing” (GO:0006396, 4 genes) (Figure 5a); molecular functions like “catalytic activity” (GO:0003824, 15 genes), “nucleic acid binding” (GO:0003676, 5 genes), and “transporter activity” (GO:0005215, 1 gene) (FDR < 0.05) (Figure 5b); and cellular components like “cell parts” (GO:0044464, 9 genes), “membrane systems” (GO:0016020, 4 genes), and “cell junctions” (GO:0030054, 2 genes) (Figure 5c).

KEGG pathway analysis reveals significant enrichment in “aminoacyl-tRNA biosynthesis” (ko00970, *p* = 1.2 × 10^−6^) and “glycolysis/gluconeogenesis” (ko00010, *p* = 0.003) (Figure 5d). Notably, GH714_033616 (POPTRDRAFT_821063) encodes an aminoacyl-tRNA synthetase involved in translation regulation; GH714_020134 (PFK3) and GH714_020079 (PFK4) are phosphofructokinases regulating sugar metabolism; GH714_002035/OXP1 is significantly enriched in glutathione metabolism (*p* = 0.049), suggesting a role in oxidative stress regulation.

### 2.5. Verification of SNP Molecular Markers

**Figure 6:**
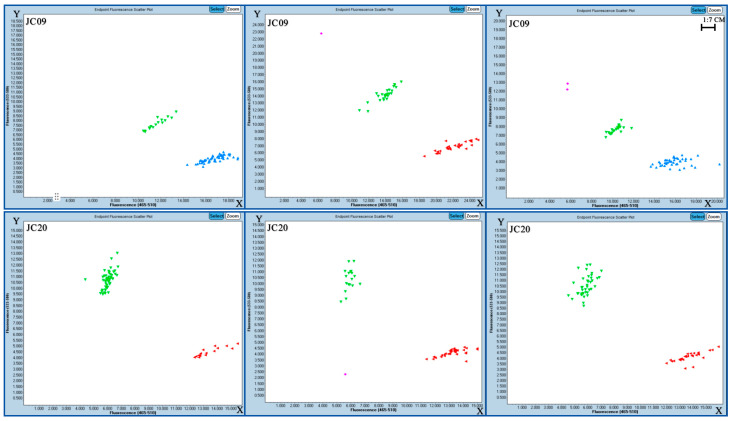
Genotyping results of JC09 (**upper**) and JC20 (**lower**). Each point represents a accession identification result, where the reading closest to the X-axis is labeled as X (marked in red or blue), the reading closest to the Y-axis is labeled as Y (marked in green), the reading between the two is labeled as XY, and the pink ones indicate reading errors.

Through screening, two molecular markers were ultimately identified for girth classification. Among 21 primer pairs, only two pairs (JC09 and JC20) showed good amplification efficiency. Figure 6 shows the classification amplification results of SNP markers JC09 and JC20.

**Figure 7:**
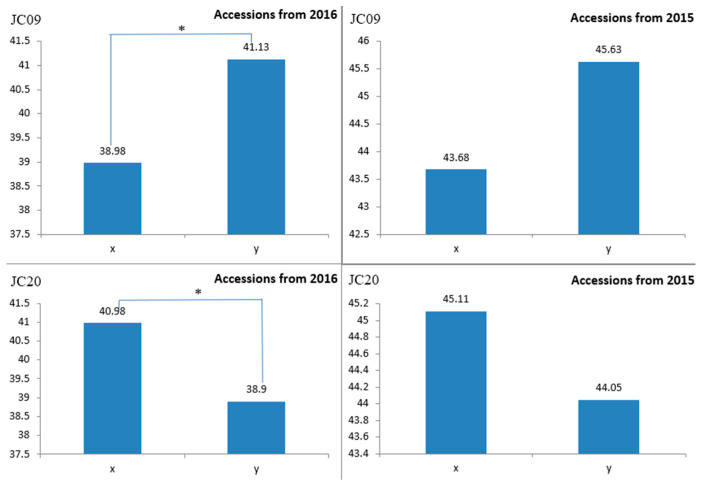
Comparison of different phenotypic categories of JC09 (**upper**) and JC20 (**lower**). * Indicates significance at the 0.05 level.

The results of the amplification of marker JC09 were matched with the girth phenotypic data of 127 accessions planted in 2016 and 66 accessions planted in 2015. Among them, the average girth of accessions with a typing result of “X” in 2016 was 40.98 cm, while the average girth of accessions with a typing result of “Y” was 38.9 cm. The average girth of the two groups of accessions differed by 2.08 cm, indicating a significant difference between the two types. For the 2015 accessions, the average girth for the “X” grouping was 45.11 cm, and for the “Y” grouping, it was 44.05 cm, with an average girth difference of 1.06 cm between the two groups. Marker JC20 showed an average girth of 38.98 cm for the “X” classification results in 2016 and an average girth of 41.13 cm for the “Y” classification results, with an average girth difference of 2.15 cm between the two groups, indicating a significant difference. In 2015, the average girth of the “X” group was 43.68 cm, and that of the “Y” group was 45.63 cm, with an average difference of 1.95 cm between the two groups (Figure 7).

## 3. Discussion

This study investigated 138 rubber tree accessions, combining phenotypic analysis, population structure analysis, and GWAS to identify key genetic loci and candidate genes related to girth. As a critical indicator of growth and latex yield, the stem girth directly impacts economic value and tapping potential. Identifying girth-related genes via molecular breeding techniques is highly significant for reducing the non-productive period, advancing tapping time, and boosting farmers’ revenue. Meanwhile, the application of genetic methods not only accelerates crop trait improvement [25] but also achieves a seamless transition from wild germplasm resource to artificial domestication [26].

Phenotypic analysis revealed significant variation in stem girth among the test accessions, and the trait followed a normal distribution (*p* = 0.143). Generally, phenotypic distributions in materials used for genome-wide association study (GWAS) should approximate a normal distribution; otherwise, phenotypic transformation is required [27]. In this study, the phenotype passed the normality test, indicating sufficient genetic diversity within the population, making it suitable for GWAS analysis [28]. Population structure analysis divided the test materials into four genetic subpopulations. Among these, Groups I and IV were genetically homogeneous, while Groups II and III exhibited admixed characteristics. The LD decay distance was 50 kb (r^2^ = 0.2), signifying the presence of a sufficient number of SNPs within this LD decay distance [29], providing a reliable foundation for association analysis.

Based on the MLM analysis, seven highly significant SNP loci (*p* < 4.4 × 10^−8^) were identified. These loci are distributed across chromosomal regions including CM021229.1, CM021235.1, and CM021239.1. Compared to previously published research on stem-girth-related traits in rubber trees [4,6,7,9], the SNP loci detected on these chromosomes are all novel. Among them, the locus at position 44,994,744 on chromosome CM021229.1 (*p* = 2.08 × 10^−9^, R^2^ = 0.254) showed the strongest association. Using KASP genotyping, primers JC09 and JC20 were developed for two well-genotyped SNP markers. The discovery of these SNP loci provides key molecular markers for stem girth traits, facilitating molecular-marker-assisted breeding.

Candidate gene mining identified a total of 27 candidate genes, mainly enriched in pathways related to metabolic regulation, signal transduction, and cell component construction. Among the identified candidate genes, the functions of some genes have been verified in previous studies. For example, PFK3 and PFK4 encode phosphofructokinase, which plays a key role in glycolysis, providing necessary energy and monosaccharide substrates for cell wall synthesis. This function has been widely confirmed in studies related to plant energy metabolism and cell wall synthesis [22]. The PHS2 gene is involved in starch metabolism, supporting cell expansion by decomposing polysaccharides, and its role in plant starch metabolism has also been clarified [23]. In addition, BACOVA_02659 encodes β-glucosidase, which affects stem mechanical strength by regulating lignin synthesis. Its role in the formation of plant mechanical strength has been reported in relevant literature [24]. However, among these four key candidate genes, PFK3/PFK4 are most likely to affect the growth of rubber tree stem girth. As key enzymes in glycolysis, PFK3/PFK4 do not directly regulate xylem development per se. However, the intermediate products generated by the glycolysis pathway (such as phosphoenolpyruvate, PEP) are the starting substrates for the phenylpropane pathway (lignin synthesis) and also serve as raw materials for fatty acid synthesis, etc. [30]. Therefore, they indirectly support the material and energy basis required for processes such as the thickening of the secondary wall of xylem cells.

Although this study identified two candidate genes (PFK3 and PFK4) and SNP molecular markers through GWAS, the experiment was conducted at a single location, and the results may therefore be influenced by environmental factors [31]. Additionally, the sample size used in this study consisted of 138 accessions. However, it is generally recommended that GWAS analyses employ a sample size of over 200 accessions to minimize the risk of false positives [32].

## 4. Materials and Methods

### 4.1. Plant Materials

Rubber tree is a tall, tropical tree in the Euphorbiaceae family, known for its trifoliate leaves and, crucially, its bark-located laticifers producing latex. Genetically, it is a diploid (2n = 36) with a large, complex, and heterozygous genome (1.5 GB) that has been sequenced (GenBank Assembly Accession GCA_010458925.1) to understand and improve rubber production traits. We selected 138 accessions of rubber tree (including 55 collected, 76 created by hybridization, and 7 introduced internationally) as the study materials, all planted in the germplasm resource nursery of the rubber tree garden at the Zhanjiang Experimental Station of the Chinese Academy of Tropical Agricultural Sciences in 1998. The accession is located in the marginal tropical monsoon climate zone. It has ample sunlight (annual sunshine duration: 1816.8 to 2073.5 h), abundant heat (annual accumulated temperature: 8309.29 to 8518.8 °C), and plentiful rainfall (annual average rainfall: 1396.3 to 1759.4 mm).

### 4.2. Phenotypic Data Collection and Analysis

In September 2022, girth of each accession was measured at 1 m above ground. Individual accession data that were missing were removed, and the average value was calculated. Phenotypic data was organized and subjected to descriptive statistics in SPSS 21, including mean, standard deviation, standard error, skewness, kurtosis, etc.

### 4.3. Genotyping by Sequencing

#### 4.3.1. DNA Extraction and Quality Testing

Genomic DNA of rubber tree leaves was extracted using a modified CTAB method [33], as follows:

1. Sample processing: Grind 1–2 g of fresh leaves to a powder with liquid nitrogen.
2. Lysis and purification: Add CTAB extraction solution (65 °C water bath for 1 h), then extract with chloroform-isoamyl alcohol (24:1) to remove protein.
3. Precipitation and washing: Precipitate DNA with isopropanol and wash three times with 75% ethanol.
4. Dissolution and quality testing: Dissolve the DNA precipitate in ddH_2_O. Check the concentration and purity (OD260/OD280 = 1.8 ± 0.2) with a BioPhotometer and confirm integrity via 1% agarose gel electrophoresis.

#### 4.3.2. Library Construction

Genomic DNA was initially digested with restriction enzymes (ApeKI, a restriction enzyme from Saixin Biotechnology Co., Ltd. (Hainan, China), is unaffected by CpG methylation and cleaves the genome uniformly), followed by the addition of barcoded sequencing adapters. Samples were then pooled to construct small-fragment libraries (250–550 bp) for PE125 paired-end sequencing.

#### 4.3.3. Genotyping by Sequencing

By digesting genomic DNA with restriction endonucleases, directly ligating sequencing adapters containing sample barcodes, and amplifying the mixture of multiple samples via PCR, fragments adjacent to the enzyme cleavage sites are simultaneously sequenced on a high-throughput sequencing instrument. Guangzhou Genedenovo Biotechnology Co., Ltd. (Guangzhou, China), was entrusted with conducting GBS (sequencing depth 30×) using Illumina high-throughput sequencing technology on the library and performing genotyping.

### 4.4. Population Stratification Analysis

#### 4.4.1. Systematically Developed Tree

Neighbor-joining trees were constructed using MEGA-X software (v7.0.26) [34] based on the SNP markers obtained after screening (model: p-distance; bootstrap: 500 times).

#### 4.4.2. Linkage Disequilibrium (LD) Decay Analysis

The PopldDecay software (v3.41) [35] was used to calculate the LD coefficient (r^2^) between pairs of markers and plot the changes as the distance increases.

#### 4.4.3. Population Structure Analysis

Population structure inference methods based on models generally assume that the markers used for the analysis are independent of each other. The Plink2 software (v1.9) [36] was used to remove one marker from each pair with r^2^ > 0.2, with a step size of 100 kb and a window size of 10 nt (markers with later physical positions were removed). The remaining SNP markers after LD filtering were used to infer the population structure and cluster them based on the K value using the Admixture software (v1.3) [37].

#### 4.4.4. Principal Component Analysis (PCA) and Kinship Analysis

In population genetics, PCA is mainly used for sample clustering analysis. Based on the filtered SNP markers, PCA analysis was performed using GCTA software (v1.93.2) [38] to obtain the variance explained by each principal component (PC) and the score matrix of the samples in each PC. Similarly, the GCTA software (v1.93.2) was used to perform a phylogenetic analysis based on the filtered markers, yielding a phylogenetic matrix of pairwise sample relationships.

### 4.5. Genome-Wide Association Study (GWAS)

GWAS was performed using the mixed linear model MLM (K) in GEMMA (v0.98.1) [39], with girth data and filtered SNPs. The population structure matrix (Q) from Admixture’s optimal K and the kinship matrix (K) from GCTA were incorporated [40,41]. Manhattan and Q-Q plots were generated using the qqman package in R (v4.5.1) to visualize GWAS results, highlight significant genetic associations, and assess statistical validity.

The expression for the GWAS model is y = Xa + Qβ + Ku + e, where y represents the phenotype, X denotes the genotype data, α stands for the genotype effect vector, Q is the fixed-effect matrix (representing population structure), β is the fixed-effect vector, K is the random-effect matrix, primarily referring to the kinship matrix, µ is the random-effect vector, and e is the residual vector. For each SNP locus, a test is conducted to check whether α is 0. The *p*-value for α being 0 indicates the degree of association between the marker genotype and the phenotype. The smaller the *p*-value, the lower the probability that α is 0, which was set as 0.05/total SNP makers (with thresholds of *p* < 0.05/225,634 = 2.22 × 10^−7^ and *p* < 0.01/225,634 = 4.4 × 10^−8^). A smaller *p*-value suggests a higher likelihood that the marker is associated with the trait.

### 4.6. Enrichment Analysis Method

Functional Annotation and Pathway Enrichment Analysis of Selected Genes (selection criteria: |log_2_FC| > 1 and adjusted *p*-value < 0.05). The specific workflow is as follows.

#### 4.6.1. Analysis Tools and Databases

The R package (v4.5.1) clusterProfiler was used to access gene annotation libraries. GO analysis covered the three main categories: biological process (GO-BP), molecular function (GO-MF), and cellular component (GO-CC). KEGG pathway analysis was based on the Kyoto Encyclopedia of Genes and Genomes database.

#### 4.6.2. Statistical Testing and Significance Assessment

Enrichment significance for functional terms/pathways was assessed using the hypergeometric test. Multiple testing correction was performed using the Benjamini–Hochberg method. A corrected false discovery rate (FDR) < 0.05 was set as the threshold for statistically significant enrichment.

#### 4.6.3. Visualization

Bar plots depicting the top 20 most significantly enriched GO/KEGG terms (sorted by ascending FDR) were generated using the enrichplot package.

### 4.7. Validation of SNP Molecular Markers

Based on the GWAS results, the chromosome (CM021239.1) containing one of the most significant SNP markers was located and selected for further analysis. We focused on the previously identified QTL interval for stem girth (CM021239.1: 1338.169 kb, 236128 bp–1574297 bp) [42] and typed the SNP variants within this region. Stem girth phenotypes were classified according to the SNP variants identified. A paired *t*-test in SPSS 21 was performed to compare the average stem girth across groups, leading to the identification of 21 SNP markers with statistically significant associations. To facilitate primer design, 100 bp flanking sequences surrounding each locus were extracted using TBtools (v2.225), and primers were designed through the SNPway platform (http://www.snpway.com/, accessed on 10 November 2023). The resulting 21 primer sets (see Supplementary Table S2) were sent to Shanghai Sangong Biotechnology Co. for synthesis.

Based on the screened significant difference SNP sites, SNP primers designed by the research team in a previous study [42] were used, and the primer sequence information is provided in Supplementary Table S2. Using the LightCycler^®^480II real-time PCR instrument (Basel, Switzerland), PCR reactions were performed in batches with the primers. Primers with better genotyping results for PCR products, as determined by the SNP genotyping software (v1.5) accompanying the LightCycler^®^480II instrument, were selected. The reaction system included 5 μL of mix reagent, a set of three KASP primer working solutions (with 0.15 μL each of primer working solutions 1 and 2 and 0.4 μL of primer working solution 3), 2 μL of DNA extract, and finally 2.3 μL of ddH_2_O to bring the reaction system to a total volume of 10 μL. The reaction program was as follows: pre-denaturation at 94 °C for 15 min, denaturation at 94 °C for 20 s, annealing and extension at 65 °C (with a gradient decrease of 0.7 °C per cycle) for 1 min for 10 cycles; denaturation at 94 °C for 20 s, annealing and extension at 57 °C for 1 min for 30 cycles.

To verify the accuracy of the SNP markers associated with stem girth in rubber tree, 66 accessions planted in 2015 and 127 planted in 2016 were selected for testing (see Supplementary Table S3). Each accession consisted of 9 plants (clonal replicates), and the average girth was calculated by measuring with a tape measure 1 m above the ground. Based on the analysis results of the LightCycler^®^480II SNP genotyping software (with X representing proximity to the horizontal axis, Y representing proximity to the vertical axis, and XY representing the area between the two), the data was categorized and matched with the stem girth phenotypic data. Significance analysis was conducted on phenotypic data across different categories using the *T*-test in SPSS. When significant differences were observed, the SNP molecular marker for rubber tree trunk girth was successfully validated.

## 5. Conclusions

This study identified loci and candidate genes related to girth growth in rubber trees through population structure analysis and GWAS. Population stratification analysis categorized the tested materials into four genetic groups. Through GWAS, at significance levels of *p* = 4.4 × 10^−8^ and *p* = 2.22 × 10^−7^, 7 and 23 SNPs significantly associated with girth were detected, respectively. Based on these loci, two SNP molecular markers were selected. Additionally, 27 candidate genes were identified. Among these, four genes (PFK3, PFK4, PHS2, BACOVA_02659) were validated. Future research should further expand the sample size, combine multi-environmental data and multi-trait analysis, deeply explore the genetic basis of rubber tree stem girth growth, and clarify the specific mechanism of action of candidate genes through functional verification experiments.

## Figures and Tables

**Table 1:** Phenotypic statistics of girth in 138 rubber trees.

Trait	Sample	Mean (cm)	SD	SE	Min (cm)	Max (cm)	Range (cm)	Skew	Kurtosis	CV	GDI	KS Test
girth	138	76.13	15.57	1.33	48.5	142	93.5	1.05	1.62	0.205	4.907	0.143

SD: Standard deviation, SE: Standard error, CV: Coefficient of variation, GDI: Genetic diversity index.

**Table 2:** Linked locus information.

Chromosomes	Position	*p*	R^2^	Genes
CM021229.1	44994744	2.08 × 10^−9^	0.254	GH714_028664; GH714_028663; GH714_028652
CM021229.1	68430381	4.03 × 10^−9^	0.299	GH714_033616; GH714_033625; GH714_033634
CM021229.1	97573289	2.93 × 10^−9^	0.580	GH714_018846; GH714_018861
CM021229.1	101378399	1.55 × 10^−8^	0.279	GH714_020134; GH714_020127; GH714_020118; GH714_020116; GH714_020113; GH714_020079; GH714_020074; GH714_020070; GH714_020064; GH714_020061
CM021235.1	69150797/69150814	2.48 × 10^−8^	0.165	GH714_002023; GH714_002035; GH714_002048
CM021239.1	2245924	5.94 × 10^−10^	0.235	GH714_035167; GH714_035169; GH714_035172; GH714_035173; GH714_035177; GH714_035184

## Data Availability

Data are available from the corresponding authors on reasonable request.

## Supplementary Materials

The following supporting information can be downloaded at https://www.mdpi.com/article/10.3390/plants14162460/s1, Supplementary Table S1: Twenty-three SNP markers; Supplementary Table S2: Primer sequence information; Supplementary Table S3: The average girth of the 193 Hevea brasiliensis trees conserved at the Zhanjiang Experimental Station of the Chinese Academy of Tropical Agricultural Sciences.

## Abbreviations

The following abbreviations are used in this manuscript:

GWAS	Genome-wide association analysis
GO	Gene Ontology
KEGG	Kyoto Encyclopedia of Genes and Genomes
LD	Linkage disequilibrium
QTL	Quantitative trait locus
SD	Standard deviation
SE	Standard error
CV	Coefficient of variation
GDI	Genetic diversity index
MLM	Mixed linear model
